# DNA damage induces expression of WWP1 to target ΔNp63α to degradation

**DOI:** 10.1371/journal.pone.0176142

**Published:** 2017-04-20

**Authors:** Ji Chen, Hua Shi, Yonglong Chen, Shijie Fan, Dingyi Liu, Chenghua Li

**Affiliations:** 1 Center of Growth, Metabolism and Aging, Key Laboratory of Biological Resources and Ecological Environment of Ministry of Education, College of Life Sciences, Sichuan University, Chengdu, China; 2 Department of Medical Oncology, The Seventh People's Hospital of Chengdu, Chengdu, China; 3 Department of Laboratory Medicine, West China Second University Hospital, Sichuan University, Chengdu, China; University of California Davis, UNITED STATES

## Abstract

ΔNp63αplays key roles in cell survival and proliferation. So its expression is always tightly controlled in cells. We previously reported that DNA damage down-regulates transcription of ΔNp63αin FaDu and HaCat cells, which contributes to cell apoptosis. In the present study, we found that DNA damage induces down-regulation of ΔNp63αvia facilitating its proteasomal degradation in cell lines such as MDA-MB-231 and MCF10A. Further investigation revealed that transcription of WWP1 is stimulated by DNA damage in these cells. Knock-down of WWP1 abrogates DNA damage-induced down-regulation of ΔNp63αand partially rescues cell apoptosis. Interestingly, DNA damage may stimulate WWP1 through different mechanisms in different cell types: it up-regulates transcription of WWP1 in a p53-dependent manner in MCF10A and HEK293 cells, while miR-452 may be involved in DNA damage-induced up-regulation of WWP1 in MDA-MB-231 cells. Our study demonstrates a novel pathway which regulates ΔNp63αupon cellular response to chemotherapeutic agents.

## Introduction

p63 gene is a homologue of p53 and p73 [1–3]. Owing to alternative transcription starting and splicing, p63 encodes a series of protein isoforms, including TAp63α_~_εand ΔNp63α_~_ε. TAp63 isotype proteins contain a full and intact transactivation domain (TAD) each at their N-termini, which endows them with potent transactivities. Activation of Bax, p21, and other downstream target genes, mediated by TAp63s, results in cell cycle arrest or cell apoptosis. ΔNp63isoforms possess a shorter and incomplete N-terminal TAD each, causing defct of transactivities in them. ΔNp63αis the predominant p63 isoform, and has been reported to antagonize transactivities of p53, TAp63s and TAp73s via forming inhibitory heterogenous complexes with these proteins or competitive binding to promoters of their downstream targets [4–9]. This is consistent with evidence supporting that ΔNp63α can enhance cell survival, growth and proliferation, though it has been recently reported that ΔNp63αswitches on transcription of genes such as MKP3 to modulate cancer metastasis and cell differentiation [3, 10–16].

In our previous study, we found that ΔNp63α is a major player in DNA damage-induced cell apoptosis in two cell lines bearing mutant p53 gene, FaDu and HaCat [17]. Treatment with DNA damage drugs, doxorubicin (Doxo) and cisplatin (CDDP), induces down-regulation of ΔNp63αand cell apoptosis. This down-regulation is independent of p53 and occurs mainly at transcription level. Knock-down of ΔNp63α directly induces cell apoptosis and increases cellular sensitivity to DNA damage agents, while exotic expression of it promotes cell proliferation and confers cells resistance to DNA damage-induced apoptosis [17, 18].

In the present work, we found that DNA damage promotes protein degradation of ΔNp63αin HEK293 cells transfected with ΔNp63α, and two cell lines expressing high level of endogenous ΔNp63α MDA-MB-231 and MCF10A. Further study revealed that transcription of WW domain-containing E3 ubiquitin protein ligase 1 (WWP1) is induced by DNA damage. Knock-down of WWP1 abrogates ΔNp63αdestabilization and partially rescues cell apoptosis induced by DNA damage. Intriguingly, we found that DNA damage enhances WWP1 mRNA level in different manners in these cells: in MCF10A and HEK293 cells, DNA damage elevates p53 protein level to enhance transcription of WWP1, while in MDA-MB-231 cells, down-regulation of microRNA-452 may account for the increase in WWP1 after DNA damage treatment. Taken together, we report here a different mechanism of DNA damage-induced down-regulation of ΔNp63α: except for decreased transcription of ΔNp63αin some cells, which we have reported previously, DNA damage can also destabilize ΔNp63αvia stimulating its E3 ligase WWP1 in a p53-dependent or some other manners in some cell lines.

## Materials and methods

### Cell culture and transfection

HEK293(ΔNp63α) [18] and MDA-MB-231 cells were grown in Dulbecco’s modified Eagle’s medium (DMEM, Hyclone) supplemented with 10% fetal bovine serum (FBS, Hyclone) and 1% penicillin/streptomycin (Hyclone). MCF-10A cells were grown in DMEM/F12 media (Hyclone), supplemented with 20 ng/ml epidermal growth factor (Invitrogen), 100 ng/ml cholera toxin (Sigma), 10 mg/ml insulin (Sigma), 500 ng/ml hydrocortisone (Sigma), 1% penicillin/streptomycin (Hyclone) sulfate and 5% FBS (Hyclone). All cells were cultured at 37°C in a humidified 5% CO2 incubator.

Small interfere RNA (siRNA) for p53 (1#sip53, Santa Cruz, sc-29435; 2#sip53, Santa Cruz, sc-44218) or WWP1 (1#siWWP1, GenePharma [18]; 2#siWWP1, Santa Cruz, sc-40366) and pre-miR microRNA precursor for microRNA-452 (Ambion, PM12509) [19] were transfected with Lipofectamine 2000 (Invitrogen) as described in the manufacture’s instruction. 100 pmol of each siRNA or pre-miR was used per well of 6-well plate at 80~90% cell confluence.

### Drug treatment and MTT assay

For DNA damage treatment, cell culture media were supplemented with 0.5 μM Doxorubicin (Doxo, Sigma) or 20 μM cisplatin (CDDP, Sigma) for 48 hours, and then subject to immunoblot, MTT assay, or qRT-PCR analysis. To inhibit proteasomal degradation of cellular proteins or measure protein half life, cells were treated with 10 μM MG132 (Sigma) or 50 μg cycloheximide (CHX, Sigma), respectively.

MTT assay was performed as previously decribed [17].

### Immunoblot analysis (IB) and Quantitative Reverse Transcription-Ploymerase Chain Reaction (qRT-PCR)

Specific antibodies to p63 (4A4 mouse monoclonal antibody, Santa Cruz, Dallas, TX, USA, 1:200), actin (rabbit polyclonal antibody, Santa Cruz, 1:1000), WWP1 (rabbit monoclonal antibody, Epitomics, Burlingame, CA, USA, 1:3000), p53 (DO-1 mouse monoclonal antibody, Santa Cruz, 1:250), and N-terminal cleaved PARP1 (rabbit polyclonal antibody, Zenable, Chengdu, China, 1:8000) were used for immunoblot analysis as described previously [18].

Quantitative reverse transcription-ploymerase chain reaction (qRT-PCR) for p63 and WWP1 was performed as decribed previously [17]. The expression level of miR-452 was analyzed with TaqMan qRT-PCR kit (assay ID: 002329) and normalized to RNU48 (assay ID: 001006) according to the manufacture’s instruction and as described previously [19].

## Results

### 1. DNA damage promotes proteasomal degradation of ΔNp63α

**Fig 1:**
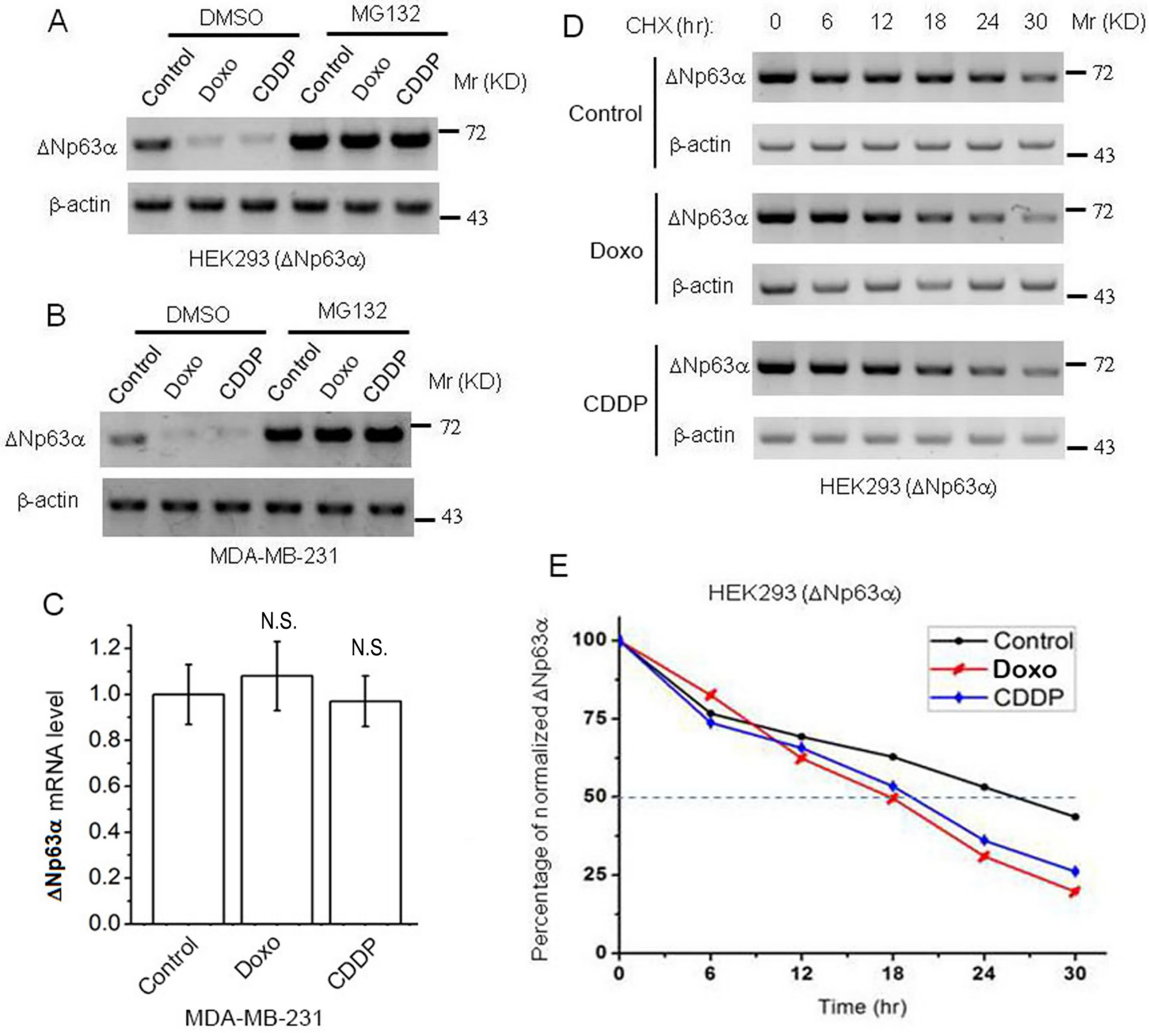
DNA damage promotes proteasomal degradation of ΔNp63α. Cells were treated with 1 μM Doxorubicin (Doxo) or 20 μM cisplastin (CDDP), plus 10 μM MG132 or its vehicle control DMSO, for 48 hours, then subject to immunoblot (IB) analysis (***A*** and ***B***) or qRT-PCR analysis (***C***). For measurement of protein half-life, cells were treated with 1 μM Doxo or 20 μM CDDP, plus 50 μg/mL cycloheximide (CHX) for indicated durations, then subject to IB analysis (***D***). Intensities of ΔNp63αbands in ***D*** were quantified with Image Lab (Bio-Rad) and normalized with β-actin bands (***E***). Expression level of ΔNp63αwas normalized to β-actin and presented as means ± standard deviations (n = 3). Analysis with Student’s *t* test reveals no significant difference (N.S.) between Doxo/CDDP-treated and control groups.

We previously reported that ΔNp63αwas significantly down-regulated in FaDu and HaCat cells after treatment with DNA damage agents, doxorubicin (Doxo) and cisplatin (CDDP); this regulation occurs mainly at mRNA level, while the half-life of ΔNp63αprotein is slightly decreased [17]. To further investigate the effect of DNA damage on protein stability of ΔNp63α, we treated a HEK293 cell line stably expressing exogenous ΔNp63α [HEK293(ΔNp63α)], which we generated previously [18], with proteasome inhibitor, MG132, in combination with either Doxo or CDPP. The results showed that either Doxo or CDDP significantly down-regulates ΔNp63αprotein level which can be dramatically restored by MG132 (Fig 1A). Similar observation was found in MDA-MB-231, which is a human breast adenocarcinoma cell line expressing endogenous ΔNp63α (Fig 1B). On the other hand, our results of quantitative reverse-transcription PCR (qRT-PCR) revealed that Doxo or CDDP does not significantly affect mRNA level of ΔNp63αin MDA-MB-231 cells (Fig 1C). Our cycloheximide (CHX) chase results revealed that protein half life of ΔNp63αis shortened by either Doxo or CDPP (Fig 1D and 1E). These results suggest that DNA damage promotes proteasomal degradation of ΔNp63α.

### 2. mRNA level of WWP1 is stimulated by DNA damage

**Fig 2:**
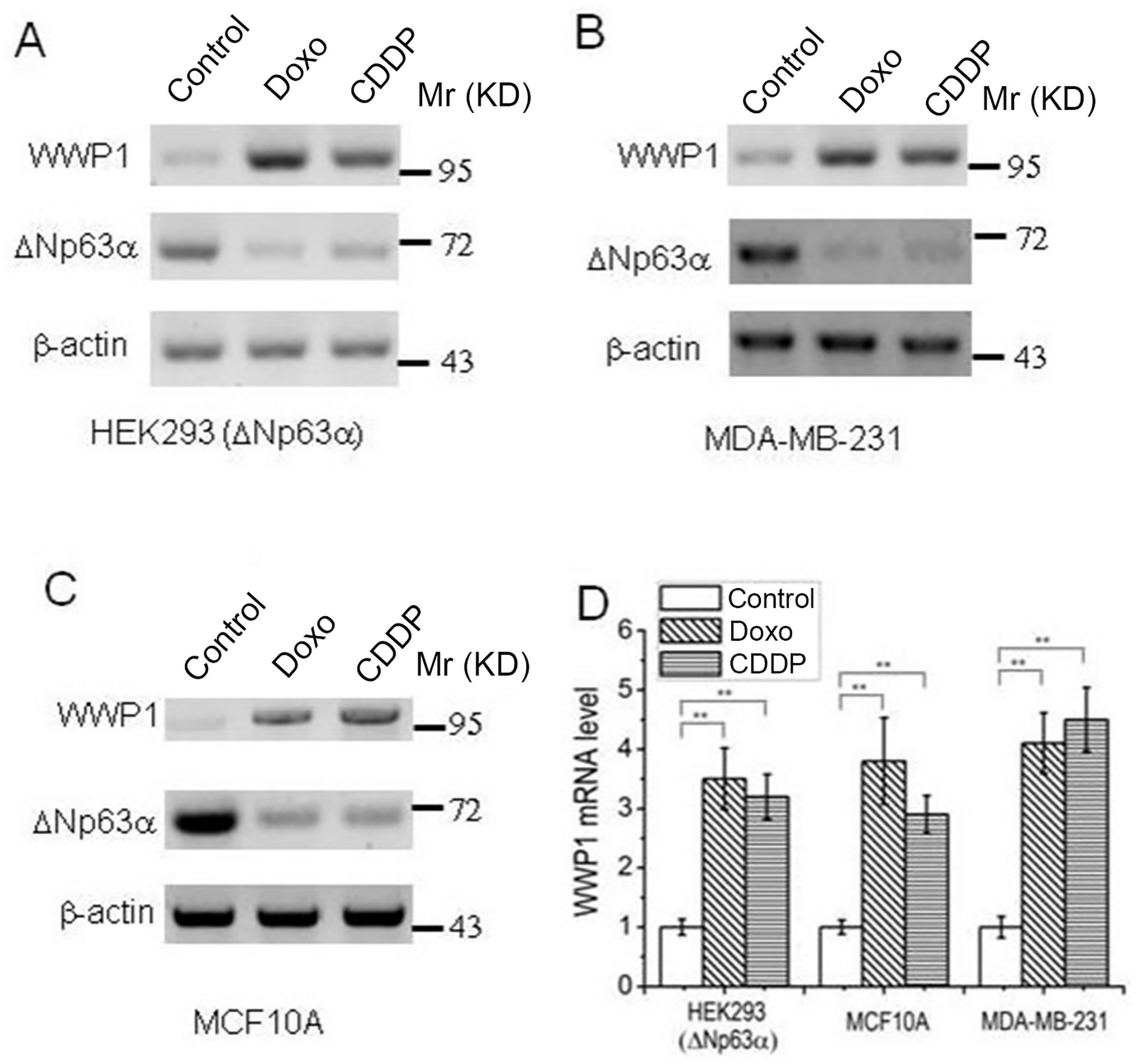
Transcription of WWP1 is induced by DNA damage. Cells were treated with 1 μM Doxo or 20 μM CDDP for 48 hours, then subject to IB analysis (***A~C***) or quantitative reverse transcription-ploymerase chain reaction (***D***). mRNA levels of WWP1 were normalized to those of GAPDH, and presented as means ± standard deviations (n = 3). *P*-values were calculated using the Student’s *t* test. **, *P*<0.01.

As an E3 ligase targeting ΔNp63αto proteasomal degradation, WWP1 was previously reported to be induced by Doxo and CDDP [20]. In line with the previous reports, we observed that either Doxo or CDDP induced up-regulation of WWP1, along with the down-regulation of ΔNp63α, in HEK293(ΔNp63α), MDA-MB-231 and MCF-10A cells (Fig 2A–2C). To study whether this up-regulation of WWP1 occurs at mRNA level, like it was reported previously [20], we measured the mRNA level of WWP1 by means of qRT-PCR. The results revealed that either Doxo or CDDP significantly increases mRNA level of WWP1 (Fig 2D). These results suggest that DNA damage may stimulate transcription of WWP1.

### 3. Knock-down of WWP1 abrogates ΔNp63αdestabilization and partially rescues cell apoptosis induced by DNA damage

**Fig 3:**
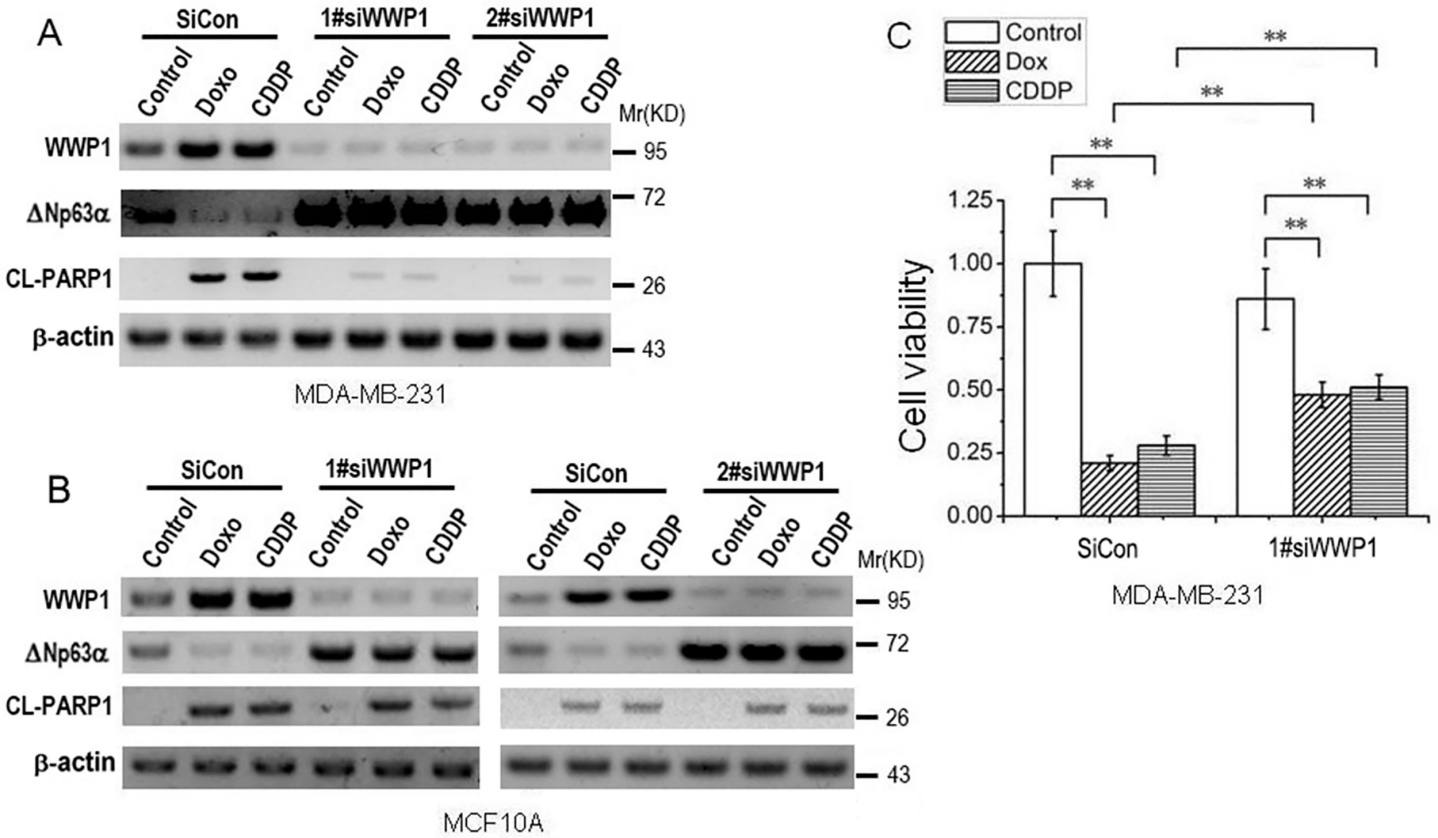
Knock-down of WWP1 abrogates ΔNp63αdestabilization and partially rescues cell apoptosis induced by DNA damage. 6 hours post transfection with siRNAs specific to WWP1 or scrambled control, cells were treated with 1 μM Doxo or 20 μM CDDP for 48 hours, then subject to IB analysis (***A*** and ***B***) or MTT assay (***C***). Cell viability was presented as means ± standard deviations (n = 3). *P*-values were calculated using the Student’s *t* test. **, *P*<0.01.

To investigate if WWP1 is involved in DNA damage-induced down-regulation of ΔNp63α we knocked down WWP1 with small interfering RNAs (siRNAs). The results showed that compared with its scrambled control (siCon), siRNA-mediated knock-down of WWP1 (siWWP1) significantly up-regulates ΔNp63αprotein level and abrogates DNA damage-induced down-regulation of ΔNp63α; concomitantly, DNA damage-induced cleavage of PARP1, which is a hall marker for cell apoptosis, is partially rescued in MDA-MB-231 cells (Fig 3A), while subtle rescue of PARP1 cleavage is observed in MCF10A cells (Fig 3B), which may be due to existence of wild-type and functional p53 in the latter cell type. The results of MTT assay confirmed the rescue effect of siWWP1 on DNA damage-induced cell death in MDA-MB-231 cells (Fig 3C). These results suggest that DNA damage-induced up-regulation of WWP1 may account for ΔNp63αdestabilization in some cell types.

### 4. p53 or miR-452 may be involved in DNA damage induced-upregulation of WWP1 transcription

**Fig 4:**
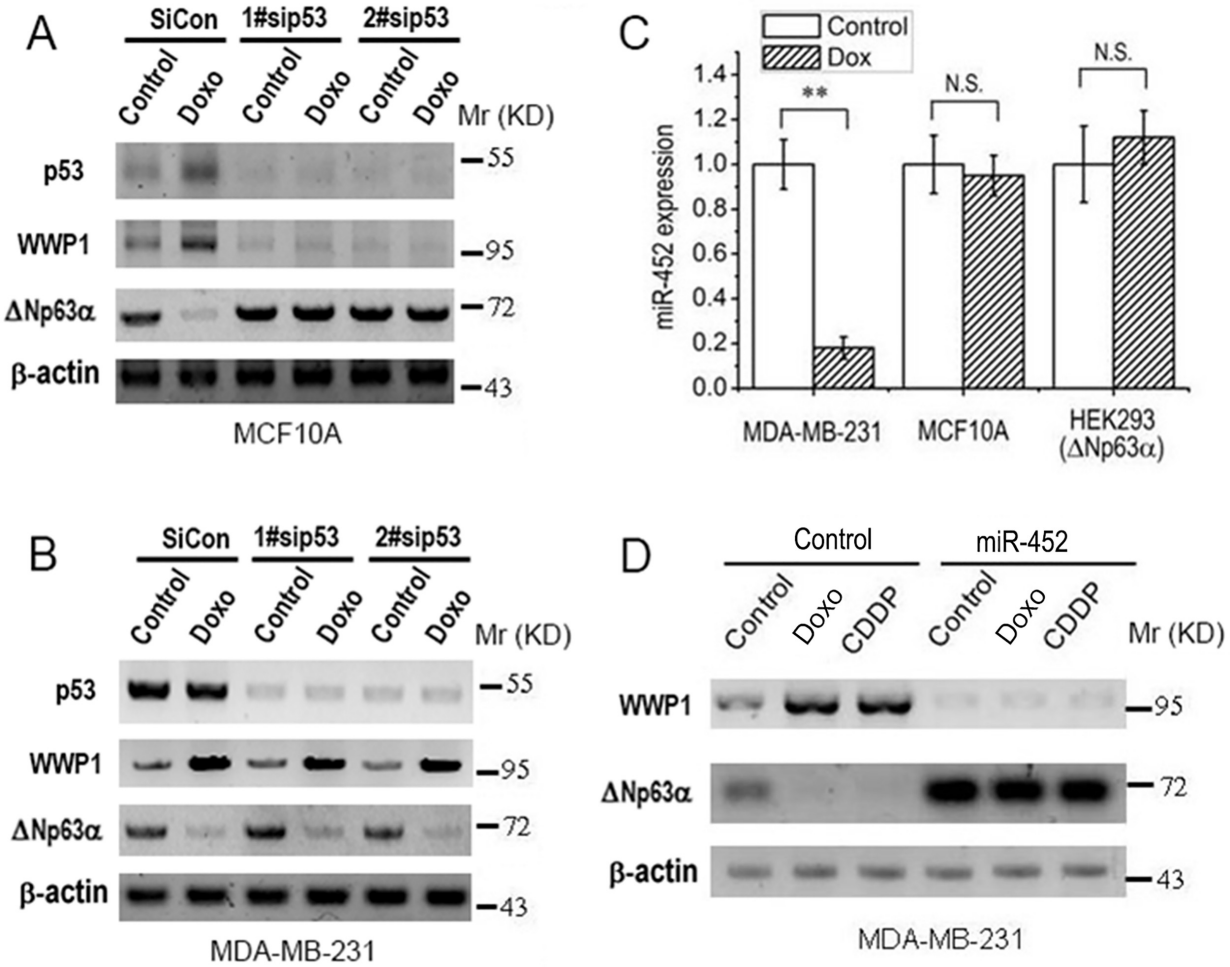
p53 or miR-452 may be involved in DNA damage induced-upregulation of WWP1 transcription. ***A*** and ***B***, 6 hours post transfection with siRNAs specific to p53 or scrambled control, cells were treated with 1 μM Doxo for 48 hours, then subject to IB analysis. ***C***, Cells were treated with 1 μM Doxo for 48 hours, then subject to qRT-PCR analysis. Expression level of miR-452 was normalized to RNU48 and presented as means ± standard deviations (n = 3). *P*-values were calculated using the Student’s *t* test. N.S., nonsignificance; **, *P*<0.01. ***D***, 6 hours post transfection with precursor for miR-452 or control, cells were treated with 1 μM Doxo or 20 μM CDDP, then subject to IB analysis.

It has been reported that DNA damage induces transcription of WWP1 via up-regulation of p53 [20]. In our system, we also found that p53 is up-regulated in MCF10A cells after treatment with DNA damage agents (data not shown). To investigate whether p53 is involved in increase of WWP1 in our system, we employed a siRNA-mediated knock-down of p53. The results reveals that in MCF10A cells, knock-down of p53 induces down-regulation of WWP1 as well as up-regulation of ΔNp63α, and abrogates Doxo-induced up-regulation of WWP1 and its effect on ΔNp63α(Fig 4A). However, in MDA-MB-231 cells, Doxo fails to increase p53 despite the up-regulation of WWP1 along with the down-regulation of ΔNp63α; on the other hand, knock-down of p53 fails to abrogate effects of Doxo on WWP1 and ΔNp63αin MDA-MB-231 cells (Fig 4B). This suggests that DNA damage may stimulate WWP1 in these two cell types in different manners.

It was recently reported that WWP1 can be directly inhibited by microRNA-452 (miR-452) [19]. To investigate whether miR-452 is involved in DNA damage-induced up-regulation of WWP1, we measured its mRNA level by means of qRT-PCR and found that after treatment with Doxo, miR-452 is significantly decreased in MDA-MB-231 cells, but not in MCF10A and HEK293(ΔNp63α) cells (Fig 4C). In another experiment (Fig 4D), we overexpressed miR-452 by transfecting MDA-MB-231 cells with its precursor and found that miR-452 significantly induces down-regulation of WWP1 as well as up-regulation of ΔNp63α; miR-452 effectively abrogates the effects of DNA damage agents on either WWP1 or ΔNp63αin MDA-MB-231 cells. These results suggest that decreased miR-452 may account for up-regulation of WWP1 as well as down-regulation of ΔNp63αinduced by DNA damage in MDA-MB-231 cells.

## Discussion

ΔNp63αis the predominant isoform of p63 gene expressed in epithelial cells [13, 21, 22]. Several E3 ligases, such as Fbw7, Itch1 and WWP1, are reported to target ΔNp63αto proteasomal degradation [20, 22, 23]. Increasing evidence shows that ΔNp63αis tightly controlled under differentiation or apoptotic conditions [5, 7, 24–26]. We previously reported that ΔNp63αinhibits cell apoptosis independently of p53 upon DNA damage: in FaDu and HaCat cells, which carry p53 mutation, ΔNp63αis significantly down-regulated upon genotoxic stress, resulting in cell apoptosis; this down-regulation of ΔNp63αinduced by DNA damage occurs mainly at mRNA level [17]. In the present study, we used a HEK293 cell line stably expressing transfected ΔNp63αand two mammary epithelial cell lines, MDA-MB-231 and MCF10A, which express high level of endogenous ΔNp63α [14, 27]. We found that DNA damage induced down-regulation of ΔNp63α also occurs in these cells. Unlike that in FaDu or HaCat cells, DNA damage-induced down-regulation of ΔNp63αin these three cell lines is attributed to facilitated proteasomal degradation of this protein (Fig 1).

Under different scenarios, E3 ligase WWP1 may execute different roles in regulating ΔNp63α. Although Peschiarroli A *et al*. reported that WWP1 mediates Lys63-linked ubiquitination of ΔNp63and regulates ΔNp63-dependent transcription but not its proteasomal degradation in primary human keratynocytes [28], evidence from Li Y *et al*. shows that in some breast cancer cell lines, particularly under DNA damage conditions, WWP1 can mediate proteasomal degradation of ΔNp63α [20]. According to Li Y’s results, DNA damage chemotherapeutic drugs, Doxo and CDDP, induce p53 expression and a concomitant up-regulation of WWP1 mRNA level in cells carrying wild-type p53; absence of wild-type p53 abrogates DNA damage-induced increase in WWP1 mRNA and protein [20]. In line with these observations, we found that either Doxo or CDDP induces significant increase in mRNA and protein levels of WWP1 (Fig 2), whose depletion can abrogate DNA damage-induced down-regulation of ΔNp63αin our system (Fig 3). In MCF10A cells, which carry wild-type p53 [14], DNA damage stimulates WWP1 expression via up-regulation of p53, of which underlying mechanism remains unknown (Fig 4A). However, in MDA-MB-231 cells, which carry mutant p53 [27], this mutant p53 does not seem to response to DNA damage; and knock-down of p53 fails to abrogate DNA damage-induced up-regulation of WWP1 in these cells (Fig 4B). On the other hand, in MDA-MB-231 but not MCF10A or HEK293(ΔNp63α) cells, DNA damage induces significant down-regulation of microRNA-452 (Fig 4C), which is recently reported as a regulatory factor of WWP1 expression [19]; and overexpression of microRNA-452 depletes WWP1 in MDA-MB-231 cells, resulting in up-regulation of ΔNp63αas well as abrogation of DNA damage-induced ΔNp63α down-regulation (Fig 4D). These results indicate that DNA damage induces WWP1 via p53 in some cells carrying wild-type p53 such as MCF10A and HEK293, or likely via miR-452 in cells carrying mutant p53 such as MDA-MB-231.

Taken together, we demonstrated that protein level of ΔNp63αcan be down-regulated in different pathways in response to genotoxic stress. On one hand, DNA damage may negatively regulate ΔNp63αvia inhibiting its transcription [17]. On the other hand, DNA damage can also induce proteasomal degradation of ΔNp63αvia stimulating WWP1 E3 ligase in different manners.
